# *In Situ* Microscopy of 2‑Dimensional Carbon Nanotube Liquid Crystals at Liquid/Liquid Interfaces

**DOI:** 10.1021/acs.langmuir.5c03535

**Published:** 2025-10-02

**Authors:** James B. Unzaga, Stephanie Oliveras Santos, Songying Li, Padma Gopalan, Arganthaël Berson, Michael S. Arnold

**Affiliations:** † Department of Materials Science and Engineering, 5228 University of Wisconsin-Madison, Madison, Wisconsin 53706, United States; ‡ Department of Chemistry, University of Wisconsin-Madison, Madison, Wisconsin 53717, United States; § Department of Mechanical Engineering, University of Wisconsin-Madison, Madison, Wisconsin 53706, United States

## Abstract

Carbon nanotubes (CNTs) must be ordered into densely aligned arrays to fully exploit their electronic properties in next-generation integrated circuits. Recent advances have shown that CNTs can accumulate and self-order at liquid–liquid interfaces, from which the CNTs can be transferred onto a substrate to create dense CNT arrays with remarkable electronic characteristics. Here, by leveraging *in situ* polarized optical microscopy, we investigate the self-assembly of CNTs at organic solvent–water interfaces and answer key questions about CNT assembly structure and formation kinetics. We find that CNTs spontaneously form liquid crystalline (LC) phases at the liquid–liquid interface with a density strongly dependent on the concentration of CNTs in the organic solvent ink. This LC behavior is robust across a range of polymer wrappers, including polyfluorenes, triblock copolymers, and polycarbazole (PCz). Polarized microscopy reveals that the resulting LC domains are polycrystalline in nature with domain size governed by the kinetics of LC formation. Additives can alter interfacial dynamics—either by promoting Marangoni flow or by enhancing CNT transport—offering an avenue to tune domain characteristics. We find that the LC domain structure formed at the interface is largely preserved upon transfer to a solid substrate, indicating that optimizing interfacial ordering is key to achieving high-quality CNT arrays for electronic applications. In cases where distortions occur during transfer, they often arise from a mismatch between the substrate translation speed and the transport velocity of the LC to the solid surface.

## Graphical abstract

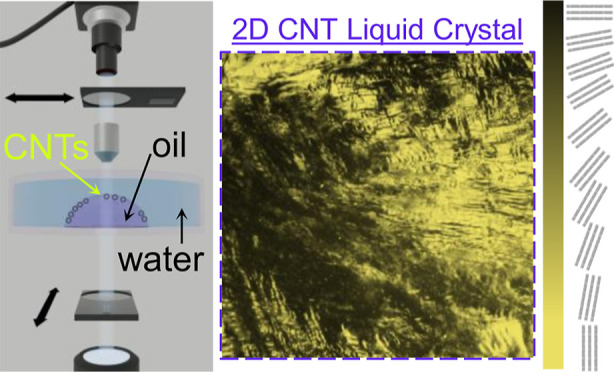

## Introduction

As state-of-the-art field-effect transistors (FETs) approach the physical scaling limit for silicon-based electronics, the development of FET channel materials that provide performance benefits beyond those achieved by scaling alone has become vital. Due to their increased current density,^1^ lower operating potential,^2^ and low-temperature solution-processability—enabling complex 3D integration schemes^3^—semiconducting single-walled carbon nanotubes (referred to here simply as CNTs) offer clear improvements over other widely used semiconductors. These transformative material properties can be harnessed only if the CNTs are ordered into dense, highly aligned arrays that are approximately one layer of CNTs thick. So far, a wide variety of approaches have been developed to fabricate such aligned arrays of CNTs, including topography-assisted vacuum filtration,^4^ dielectrophoresis,^5^ solution shear,^6,7^ DNA-directed assembly,^8^ Langmuir–Blodgett,^9^ and Langmuir–Schaefer.^10^ An especially promising approach to make wafer-scale high-performance arrays is a class of alignment techniques that utilize self-assembly at the interface between two liquids.^11–13^ These approaches leverage the accumulation of CNTs at the liquid–liquid interface, where they can dynamically reorder, sometimes assisted by tangential ink flow at the interface, before being transferred onto a substrate that is slowly pulled through the interface. For example, when CNTs or any nanoscale material^14,15^ that is sterically dispersed in an organic solvent (i.e., CNT ink) contacts water, they accumulate at the liquid–liquid interface. This behavior is consistent with observations in other nanoparticle systems, where interfacial adsorption is energetically favorable because nanoparticles that collect at the interface displace fluid, minimize interfacial area, and reduce interfacial tension.^16^

In our previous work,^13^ we inferred that CNTs form 2D nematic liquid crystals (LCs) at the liquid–liquid interface, inducing self-alignment prior to deposition onto a substrate. However, ref 13 provides no direct evidence to support this hypothesis—only that CNTs are aligned in dense arrays about one layer of CNTs thick after deposition onto the substrate. Despite the lack of direct evidence, there is theoretical support for this idea. Onsager’s rigid rod theory predicts that when rigid rods (e.g., CNTs) reach a critical bulk density, the rods form a nematic LC phase.^17,18^ Onsager’s theory can also be adapted to consider rods confined to a 2D area like CNTs confined to a liquid–liquid interface. The CNTs used here and in previous works (e.g., ref 13) can be considered rigid because their diameter and length (1.5 and 500 nm, respectively) are much less than their persistence length of more than 10 μm.^19^ For rigid rods of this size, Onsager’s theory predicts that nematic behavior emerges at an areal density of 28 CNTs μm^–2^ (see Calculation S1 in the Supporting Information).^20^

Although this indicates that an LC transition is possible, it does not provide insight into how CNT LCs might behave. It is equally plausible that the LC exists across the entire interfacial area or forms only near the triple contact line, where the substrate meets the interface. When a 2D-LC of CNTs does form at the liquid–liquid interface, it is also not clear if the arrangement of CNTs at the interface is preserved as the CNTs are transferred to the substrate. If so, then optimizing the degree of CNT ordering at the liquid–liquid interface will be important for increasing the quality of CNT ordering on the substrate. Without the ability to observe CNT LCs at the liquid–liquid interface, these questions and several others about the 2D-LC grain size, formation kinetics, and deposition mechanism remain unanswered.

Cross-polarized optical microscopy (POM) is a characterization technique previously used to study the interfacial ordering of common molecular LCs such as sodium dodecyl sulfate^21^ and 4′-pentyl-4-biphenylcarbonitrile.^22,23^ In POM, unpolarized light passes through a polarizer and the birefringent CNT array, which rotates its polarization. A second polarizer, set ∼90° to the first, filters the transmitted light before camera detection, giving contrast that reflects CNT density, order, and alignment (see Figure 1A and the Sessile Droplet LC Formation and POM Imaging section for more details).

**Figure 1:**
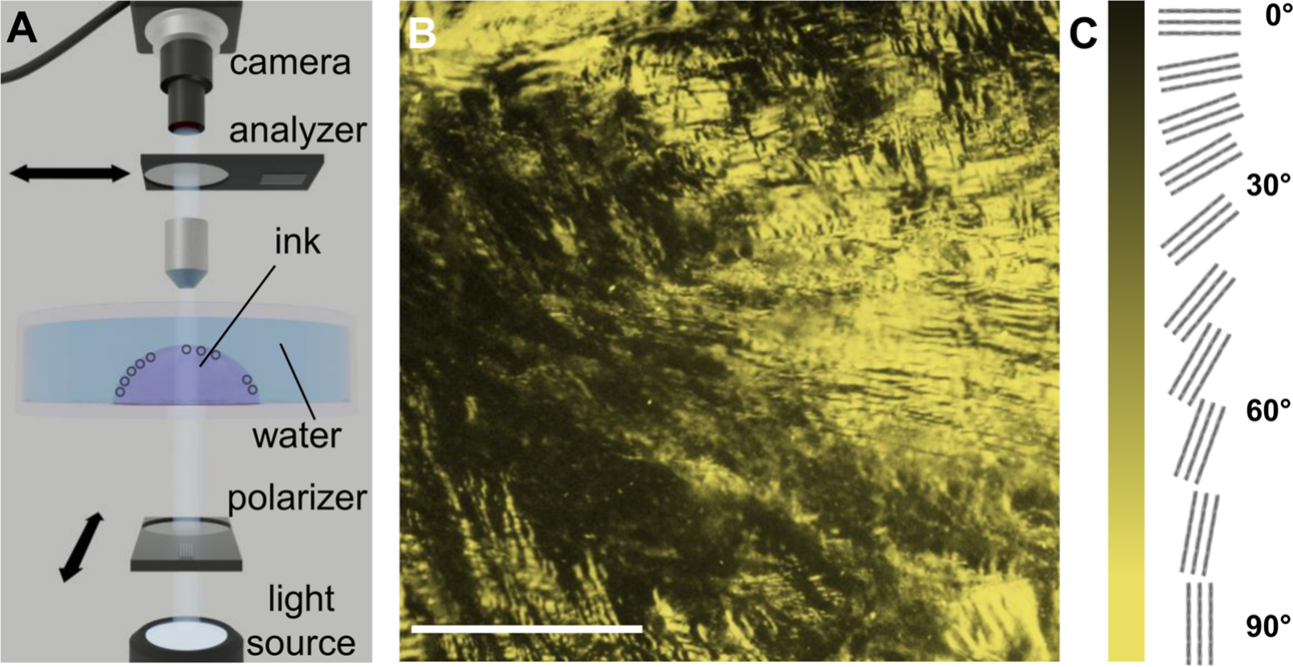
(A) Experimental apparatus for the *in situ* characterization of 2D CNT LCs. (B) POM image of a polycrystalline CNT LC at the liquid–liquid interface grown from 80 μg mL^–1^ ink. The scale bar in (B) is 500 μm. (C) Schematic illustrating the relationship between signal color and CNT orientation in POM data.

Here, by leveraging POM, we verify and study the formation of 2D-LCs of conjugated polymer-wrapped CNTs at a chloroform–water liquid–liquid interface similar to that used by Jinkins et al. We investigate how CNTs self-assemble at such interfaces and find that they spontaneously form polycrystalline LC domains whose size depends on the kinetics of formation and the CNT concentration. Additives can influence interfacial dynamics and transport, enabling control over the domain structure. These LC patterns are largely retained upon transfer to a substrate, with distortions mainly arising from mismatches between the substrate speed and LC transport.

## Experimental Section

### Polyfluorene (PFO-BPy) CNT Sorting

Semiconducting carbon nanotubes are isolated from arc-discharge nanotube soot (Sigma-Aldrich, #698695) using a polyfluorene derivative polymer wrapper, poly­[(9,9-dioctylfluorenyl-2,7-diyl)-*alt*-*co*-(6,6′-{2,2′-bipyridine})] (PFO-BPy) (*M*_W_ 74 kDa, OMI153UV, Montreal Optoelectronics Inc., Quebec, Canada), at a 1:1 mass ratio. PFO-BPy is first dissolved in anhydrous toluene (2 mg/mL) and is then combined with the nanotube soot. This solution is sonicated at 40% amplitude (∼200 W) for 10–30 min using a horn-tip ultrasonicator (Sonic Dismembrator 500, Thermo Fisher Scientific, Waltham, MA). The sonicated dispersion is centrifuged at 300,000*g* (corresponding to 41,000 rpm, average rotor radius of 11.3 cm) for 10 min using a Sorvall WX ultracentrifuge (TH-641 rotor, Thermo Fisher Scientific) to remove undispersed nanotubes and amorphous carbon. The supernatants are concentrated from 300 to 60 mL total volume using a rotary evaporator. This concentrated solution is further purified via iterative centrifugation (24 h per cycle) and redispersion in fresh toluene until the PFO-BPy:nanotube ratio reaches ∼1:3 by mass (3–4 cycles) as determined optically.^1^ The purified CNT dispersion is redispersed in chloroform (stabilized with ethanol, Thermo Fisher Scientific, #C606SK-1, or stabilized with amylene, Sigma-Aldrich, 472476-1L) to create the final ink. The nanotube concentration is determined optically using a UV–vis–NIR spectrophotometer from the S_22_ transition.^13^

### Triblock Copolymer CNT Sorting

Semiconducting carbon nanotubes are isolated from arc-discharge nanotube soot (Carbon Solutions, AP-SWNT) using a synthesized triblock copolymer PS_139_-*b*-PFO_20_-*b*-PS_139_ at a 1:1 mass ratio, where PS denotes polystyrene and PFO denotes poly­(9,9-dioctylfluorene). The triblock copolymer is first dissolved in anhydrous toluene (1 mg/mL), then combined with the nanotube soot. This solution is sonicated at 40% amplitude (∼200 W) for 10 min using a horn-tip ultrasonicator (Sonic Dismembrator 500, Thermo Fisher Scientific, Waltham, MA). The sonicated dispersion is centrifuged at 300,000*g* (corresponding to 55,000 rpm, average rotor radius of 11.3 cm) for 10 min using a Sorvall WX ultracentrifuge (Thermo Scientific Sorvall MX120+) to remove undispersed nanotubes and amorphous carbon. The supernatant is then filtered using 5 μm PVDF membranes (Millex, SLSV025LS) to remove any large aggregates. The supernatant is collected, followed by three cycles of 3 h centrifugation at 3 × 100,000*g* (Thermo Scientific Sorvall MX120+) to remove excessive free polymer in the dispersion. The pellets are collected and redispersed in chloroform. The nanotube concentration is determined optically using a UV–vis–NIR spectrophotometer from the S_22_ transition.^1,37^

### Polycarbazole (PCz) CNT Sorting

Semiconducting carbon nanotubes are isolated from arc-discharge nanotube soot (Sigma-Aldrich, #698695) using a polycarbazole derivative polymer wrapper, poly­[*n*-(1-octylnonyl)-9*H*-carbazole-2,7-diyl] (PCz) (*M*_W_ 63 kDa, 21C012A1, American Dye Source Inc., Quebec, Canada), at a 1:1 mass ratio. PCz is first dissolved in anhydrous toluene (2 mg/mL) and then combined with the nanotube soot. This solution is sonicated at 40% amplitude (∼200 W) for 10–30 min using a horn-tip ultrasonicator (Sonic Dismembrator 500, Thermo Fisher Scientific, Waltham, MA). The sonicated dispersion is centrifuged at 300,000*g* (corresponding to 41,000 rpm, average rotor radius of 11.3 cm) for 10 min using a Sorvall WX ultracentrifuge (TH-641 rotor, Thermo Fisher Scientific) to remove undispersed nanotubes and amorphous carbon. The supernatants are concentrated from 360 to 60 mL of total volume using a rotary evaporator. This concentrated solution is further purified via iterative centrifugation (24 h per cycle) and redispersed in fresh toluene until the PCz:nanotube ratio reaches ∼1:3 (3–4 cycles) as determined optically. The purified CNT dispersion is redispersed in 1,1,2-trichloroethane (Sigma-Aldrich, 466212-25 ML) to create the final ink. The nanotube concentration is determined optically using a UV–vis–NIR spectrophotometer from the S_22_ transition.

### Sessile Droplet LC Formation and POM Imaging

In a POM experiment, an unpolarized light source is passed through a linear polarizer (i.e., the polarizer). Due to their birefringence, ordered CNT arrays rotate the polarization of transmitted light.^38^ The transmitted signal is filtered by using a second linear polarizer (i.e., the analyzer) rotated roughly 90° from the polarization of the light source and then detected with a camera. The image contrast at the camera is determined by the density of CNTs, their degree of ordering, and their direction of alignment with respect to the polarizer and analyzer orientations.

In POM experiments, 100 μL of chloroform carbon nanotube ink (1–240 μg mL^–1^) is manually injected, using a syringe (Hamilton, 81320) with a 24-gauge needle, into the center of a 100 mm borosilicate glass Petri dish containing deionized water (resistivity, ∼18 MΩ). The interface is then imaged using a Nikon OPTIPHOT-2 transmission microscope (5× objective) modified with a pair of nearly perpendicular linear polarizers (0° and 89/91° orientation). Still images are captured with a high-resolution 14MP digital camera (AmScope, MU1403) mounted in the eyepiece tube. Images are postprocessed to enhance contrast and focal stacked to improve the depth of field using Zerene Stacker (Pmax). Once optimized, the still images are then recolored using a recoloring algorithm detailed in Calculation S2. Brightness and contrast values can be found in Table S1.

### Particle Tracking Velocimetry

Particle tracking is performed using TracTrac (open-source MATLAB-based software), analyzing 300 frames per experiment. Dark particles are detected using a Difference of Gaussians (DoG) filter with subpixel Gaussian refinement. Noise filtering (5 px) is applied, and particle motion is modeled as unsteady with a 1-frame delay. Velocity fields are visualized using average velocity mode, and outliers are filtered based on a 2 standard deviation threshold.

### Visualized CNT-LC Transfer

For studies of CNT-LC transfer from the liquid–liquid interface to substrate, a custom-made 10 mL beaker is created by bisecting a 20 mL-vial and flame-polishing the beaker’s edge. First, 4 mL of deionized water (resistivity ∼18 MΩ) is added to the beaker. Then, 8 mL of 10 μg mL^–1^ CNT-chloroform ink is manually added via a syringe needle in contact with the bottom of the beaker. The excess water is then pipetted out of the beaker to prevent overflowing. This process results in a layer of water overlying a layer of CNT chloroform ink in the beaker with a fairly flat interface.

This beaker is then placed onto a glass platform mounted to a substrate insertion motor (Thorlabs, MTS25-Z8). A cleaved #1 glass coverslip (Fisherbrand, 12-541-057) is used as the substrate. Substrates are hydroxylated through UV–ozone exposure for 1 h. The substrates are then functionalized with hexamethyldisilazane (HMDS; Thermo Scientific, L16519-AC) in a vapor chamber for 3 h. The HMDS-treated substrate is placed onto the motor fixture and inserted at 45° from normal, from the top-down, into the interface at a 0.18 to 100 mm min^–1^ velocity. This apparatus is placed below the objective of a cross-polarized transmission microscope for video capture. After focusing on the triple contact line, the deposition is then recorded at 25 fps (4096 × 3286 resolution), or time lapse imaging is performed at 0.1 fps. Once optimized, the videos are then recolored using a recoloring algorithm detailed in Calculation S2. Brightness and contrast values can be found in Table S1. After the LC was transferred to the substrate, the water is removed from the beaker. If the substrate is removed from the beaker by withdrawing it through the water layer, rope-like bundles of CNTs are created. Once the substrate is removed, it is immediately submerged in 2-propanol and dried in a stream of nitrogen.

## Results and Discussion

The POM apparatus used for directly characterizing and studying the interfacial assembly of CNTs is shown in Figure 1A. To check for interfacial assembly, CNT ink is manually injected into a water-containing Petri dish. A droplet of ink forms at the bottom of the Petri dish, submerged beneath the water. A liquid–liquid interface forms at the surface of the droplet. A typical droplet is 0.25 cm in diameter with a CNT ink concentration of 80 μg mL^–1^, within the range commonly used in previous experiments.^13^

Within 20 s of injecting the CNT ink, a birefringent film forms at the liquid–liquid interface (Figure 1B,C). Changing the angle of the analyzer from ∼89° to ∼91° relative to the source polarizer flips the birefringent contrast (Figure 2A,B), as expected for aligned CNTs.^24^ No contrast is observed in control experiments lacking CNTs, in which droplets of chloroform or chloroform containing the wrapping polymer poly­[(9,9-dioctylfluorenyl-2,7-diyl)-*alt*-*co*-(6,6′-{2,2′-bipyridine})] (PFO-BPy) are injected into the water bath (Figure S1), confirming that the birefringent contrast in Figure 2A,B originates from the CNTs.

**Figure 2:**
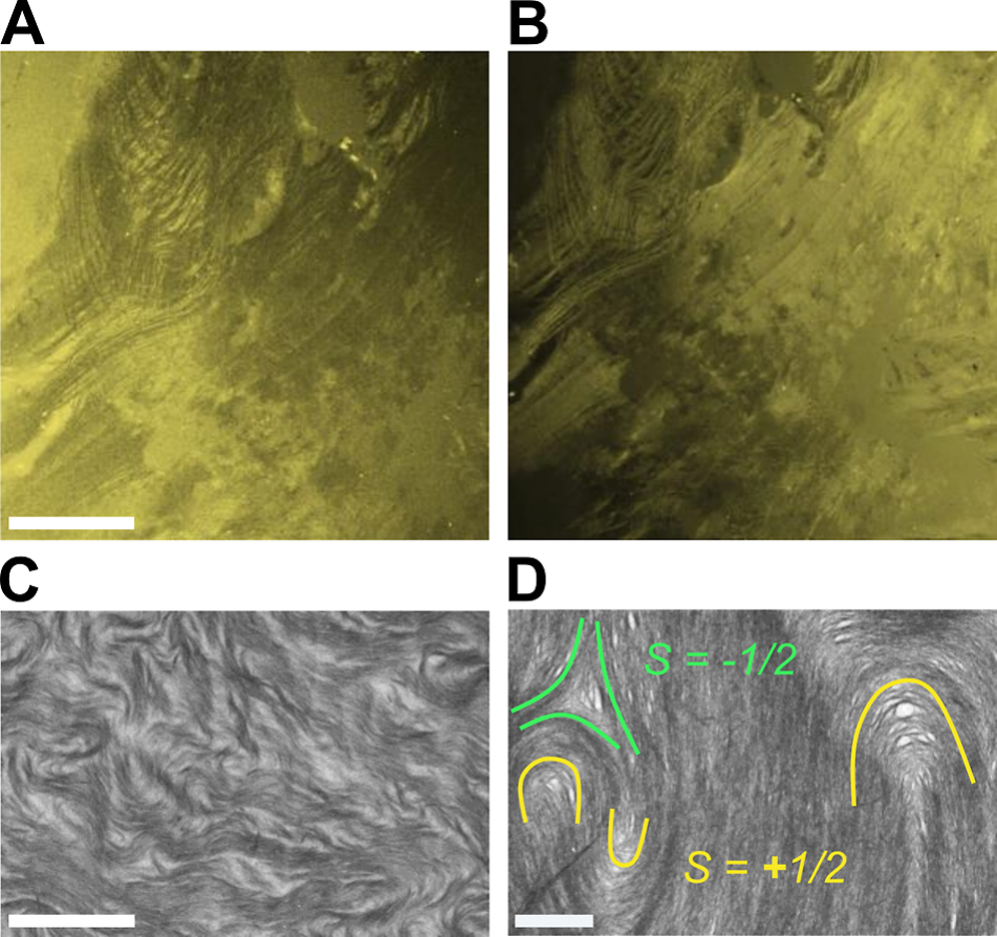
(A,B) Normal and reverse POM polarizations of a Langmuir–Schaefer deposited CNT film using 10 μg mL^–1^ ink; 500 μm scale bar. (C) SEM of the CNTs forming a schlieren texture; 20 μm scale bar. (D) SEM of the deposited CNT film with −1/2 and +1/2 disclinations; 1 μm scale bar.

To further confirm that the birefringent contrast in Figure 2A,B originates from the CNTs and that 2D-LCs of CNTs exist at the liquid–liquid interface, a glass slide is lowered into the water bath parallel to the apex of the droplet until the glass slide makes contact with the droplet formed from CNT ink. The film at the liquid–liquid interface transfers to the glass slide. This Langmuir–Schaefer-like transfer technique deposits the film onto the slide while minimizing in-plane deformations during transfer. Preserving the structure of the film allows for the characterization of the LC domains using higher-resolution microscopy techniques such as scanning electron microscopy (SEM). SEM imaging confirms that the deposited film consists of an LC of CNTs. The deposited CNT film exhibits inhomogeneities that resemble a schlieren texture—an ordering pattern commonly observed during nematic LC formation^25^—as seen in Figure 2C. Closer examination reveals that the CNTs form characteristic LC disclinations (Figure 2D), further confirming that CNT self-assembly is governed by LC behavior.

2D CNT LCs are observed to form at the liquid–liquid interface at CNT ink concentrations as low as 5 μg mL^–1^ and as high as 240 μg mL^–1^, with more CNTs accumulating at the interface as the CNT ink concentration increases (Figure S2). 2D CNT LCs also form at the interface when using alternative polymer wrappers (Figure S3), including polystyrene–polyfluorene–polystyrene triblock copolymers.^26^ 2D LCs of CNTs wrapped by poly­[9-(1-octylnonyl)-9*H*-carbazole-2,7-diyl] (PCz) also form at the interface of 1,1,2-trichloroethane and 2-butene-1,4-diol (Figure S3), the solvent system used in dimension-limited self-alignment (DLSA).^11^ These results show that the accumulation and ordering of CNT LCs at a liquid–liquid interface are not unique to PFO-BPy (the wrapper used for the remainder of this study) and are not limited to the chloroform–water system (the pair of solvents used for the remainder of this study).

Previous work on liquid–liquid interfacial self-assembly found that additives to the CNT ink affect the ordering of CNTs deposited on the substrate, with ethanol being particularly important since it is present in some chloroform formulations as a stabilizer.^12,13^ 2D-LCs of CNTs are found to readily form at the liquid–liquid interface using chloroform without ethanol (instead using amylene as a stabilizer), in chloroform stabilized with 1.4%, 5%, and 10% ethanol added by volume. However, the addition of ethanol strongly affects the size of the LC domains. Increasing the ethanol content within the ink from 1.4% (Figure 3A) to 5% (Figure 3B) produces larger LC domains. Further increasing the ethanol content within the ink to 10% by volume does not further increase the domain size. Ethanol increases the domain size by influencing the dynamics of 2D-LC formation, as further characterized below.

**Figure 3:**
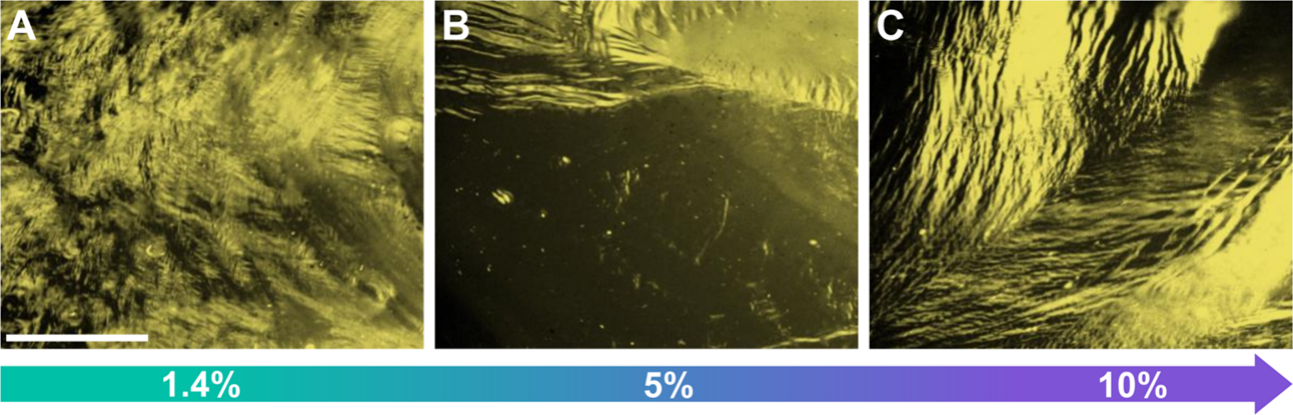
(A–C) POM images of CNT LCs grown from 80 μg mL^–1^ ink at 1.4% and 5% ethanol, respectively. The scale bar in (A) is 500 μm and applies to all images.

Video S1 shows sequential POM images capturing the dynamics of a 2D-LC of CNTs forming at the liquid–liquid interface with an ink containing 1.4% ethanol. Still images extracted from the movie at 20, 25, and 30 s are shown in Figure S4. Within 30 s, most of the interface is covered by a 2D polycrystalline LC that is not appreciably evolving with time.

In contrast, Video S2 shows sequential POM images of a 2D-LC of CNTs forming at the liquid–liquid interface by using an ink containing 5% ethanol. Still images extracted at 10, 50, 76, and 122 s are shown in Figure 4A–D. In contrast to Video S1, in Video S2 with 5% ethanol, the liquid–liquid interface is not completely covered with a 2D-LC of CNTs until ∼110 s after the injection of the ink into the water bath. In the 5% ethanol experiment, birefringent domains of CNTs are observed near the droplet apex at the liquid–liquid interface almost immediately after the droplet is introduced; for example, see the birefringent contrast in Figure 4A, Video S2 (8–12 s), and Figure S5A. However, these domains are rapidly swept to the edges of the droplet; for example, see Video S2 (12–40 s) and Figure S5B,C. Following this, 2D-LC domains slowly grow from the edges of the droplet up toward the droplet apex over a duration of ∼100 s. These differences yield larger 2D LC domains.

**Figure 4:**
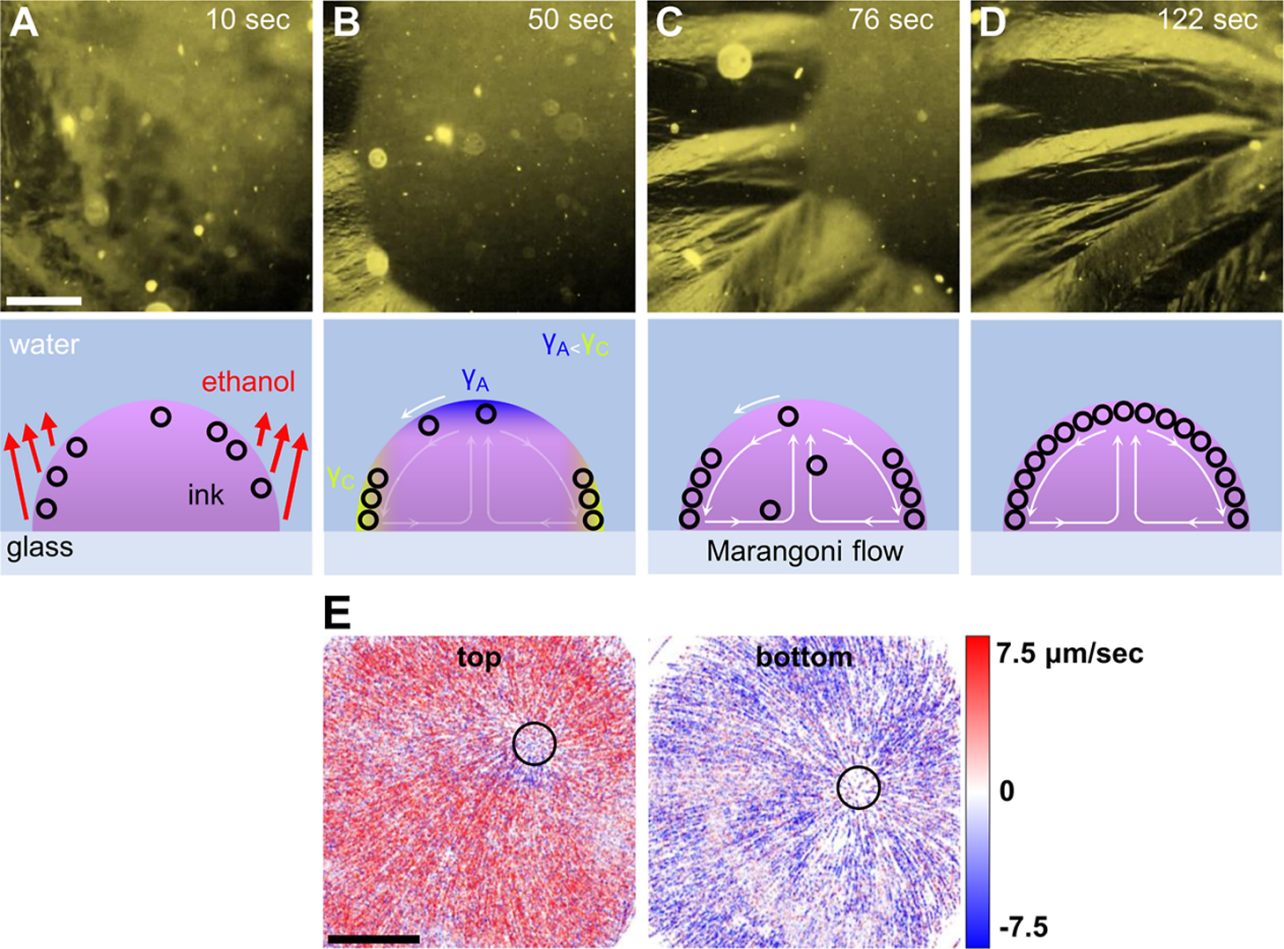
POM images of the left side of an ink droplet (with 5% ethanol) with an accompanying schematic of different stages of LC growth: (A) During the rapid nucleation of LC at the liquid–liquid interface following the introduction of ink into water. These LC domains are swept to the droplet edges. (B) Pinning of LC along the contact line at droplet edges (left side of the image), beginning of inward growth. (C) Inward growth of LC directed by Marangoni flow. (D) Completely grown large domain LC directed by Marangoni flow. Full evolution shown in Video S2. (E) Particle tracking velocimetry color maps of radial flow within a sessile ink droplet near the top and bottom of the droplet. Center of droplet flow outlined. The scale bar in (A) is 500 μm and applies to (A) to (D). The scale bar in (E) is 500 μm.

Higher ethanol content causes several changes. First, ethanol induces mass transfer across the interface due to its miscibility in both solvents. This mass transfer drives the convection of CNTs to the interface. The second effect induced by a higher ethanol content is a Marangoni flow. If surface tension is lower at the apex of a sessile droplet than at the contact line (where the liquid–liquid interface meets the substrate at the droplet edge), Marangoni flow is induced along the interface from apex to edge.^27–29^ Because of a thinner diffusion boundary layer and a larger effective surface area at the contact line, this region depletes ethanol faster than the apex. This relative decrease in ethanol also increases the surface tension along the contact line, inducing flow along the interface from the apex. In our case, the solutal Marangoni flow transports CNT LC domains formed at the apex toward the droplet edges. If the flow is sufficient (e.g., at 5% ethanol), LC domains accumulate at the droplet edges and then grow inward. This process (depicted in the schematics in Figure 4A–D) leads to larger domains, possibly because the orientation of the CNTs is set by the flow or because of a CNT–CNT or CNT–substrate templating effect at the liquid–liquid interface–substrate contact line.

Particle tracking velocimetry (PTV) directly observes Marangoni flow patterns and quantifies the flow speeds. PTV tracer particles are prepared by grinding silicon into a fine powder and adding it to CNT ink prior to underwater injection. A radial velocity map for the top of the droplet (left) and the bottom of the droplet (right) can be found in Figure 4E. Near the droplet top, particles flow radially outward, while at the bottom, flow is radially inward—supporting the recirculating Marangoni flow hypothesis depicted in Figure 4C,D. The average radial flow speed is 260 μm s^–1^ in the 5% ethanol ink. An example PTV movie is provided in the Supporting Information in Video S3. Marangoni flow is also present when using the 1.4% ethanol ink, but the flow velocity is slower, averaging 190 μm s^–1^ in the radial direction. The observation that CNT LC domains slowly grow from the droplet edges, inward, at 5% ethanol suggests that the CNTs that accumulate at the liquid–liquid interface are swept from the apex to edges, carried by Marangoni flow. The fact that this slow growth from the edges is not observed at 1.4% ethanol may indicate that a threshold velocity is needed to transport LC domains to droplet edges. Because these observations are made in sessile droplet geometries, it remains uncertain how directly they extend to other configurations, such as planar liquid–liquid interfaces or continuous-flow setups. The extent of Marangoni-driven transport may depend on boundary conditions, interface curvature, and confinement. It is not yet clear whether similar transport would occur at planar liquid–liquid interfaces or in continuous-flow systems. Future work will be needed to assess the extent to which these findings can translate to other geometries.

Next, the transfer of 2D-LCs of CNTs from the liquid–liquid interface to a translating substrate is studied directly via POM. Current liquid–liquid interfacial CNT deposition techniques involve withdrawing and pulling a substrate through a CNT-laden interface. It remains unknown whether CNT ordering on the substrate correlates with CNT ordering at the liquid–liquid interface. For these experiments, ∼8 mL of CNT ink in chloroform is added to a Petri dish 2.8 cm in diameter. ∼2 mL of water is then layered on top of the chloroform. A glass substrate is inserted into the beaker at a 45° angle from the vertical, emulating previous CNT depositions.^13^ The structure and dynamics of the CNT LCs at the liquid–liquid interface and their transfer to the substrate are imaged in real time in a POM microscope, as depicted in Figure 5A. Figure 5B–D shows three sequential POM images taken as the substrate is pushed into the beaker at a velocity of 0.18 mm min^–1^. The lower-right portion of the image shows the liquid–liquid interface. The observed POM contrast here indicates the presence of a 2D-LC of CNTs. As the substrate is pushed into the interface, features present in the 2D-LC translate and move toward the substrate. An example feature is highlighted by the dashed box in Figure 5B. This feature feeds into the triple contact line formed between the liquid–liquid interface and the substrate in Figure 5C and then completely transfers onto the substrate (the upper-left portion of the image) in Figure 5D. Throughout the translation and transfer, the feature retains its original form. Video S4 shows the full transfer and deposition process.

**Figure 5:**
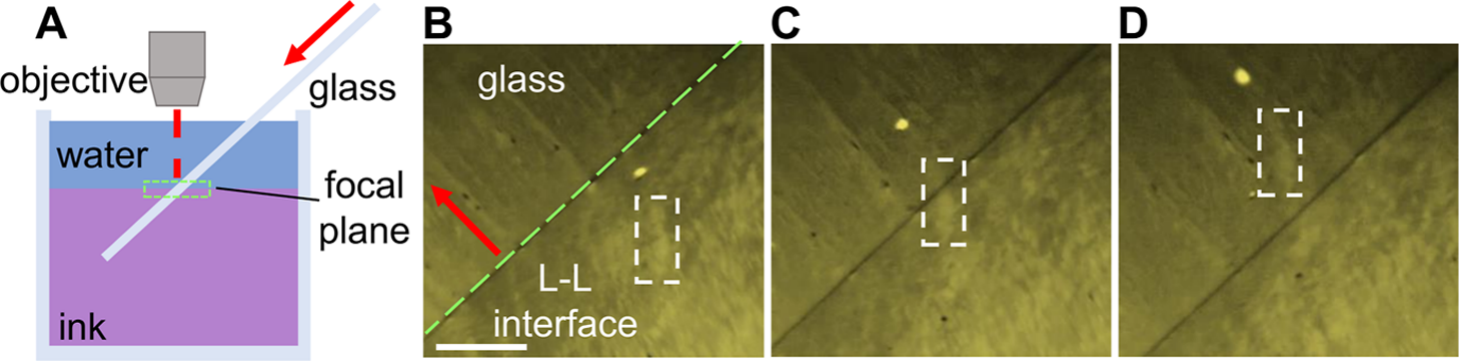
(A) Apparatus used to characterize CNT deposition onto the substrate *in situ*. The red arrow indicates direction of substrate motion. (B) An outlined feature of the LC at the liquid–liquid interface. The contact line is highlighted with a green dashed line. (C) The feature during transfer from the liquid–liquid interface to the substrate at the contact line. (D) The feature after deposition onto a glass substrate. The scale bar in (B) is 250 μm and applies to all images.

However, not all features observed at the liquid–liquid interface transfer faithfully onto the substrate. The LC undergoes various distortions during deposition, including domain streaking, domain rotation (orthogonal to the axis of substrate tilt), and film tearing as cataloged in Figure 6A–C. In some instances, particles become momentarily trapped at the contact line (see Figure 6A). Long streaks of CNTs with the same POM intensity/color are observed on the substrate in the trail of each particle, indicating that particles induce CNTs to reorder along the direction of transfer from the liquid–liquid interface to the substrate. The inset in Figure 6A is a characteristic SEM image of one of these streaks, showing that they are primarily composed of CNTs aligned along the direction of substrate travel. Observed domain rotation and film tearing (Figure 6B) can be attributed to a velocity mismatch between the LC feed rate into the triple contact line and the faster moving substrate.

**Figure 6:**
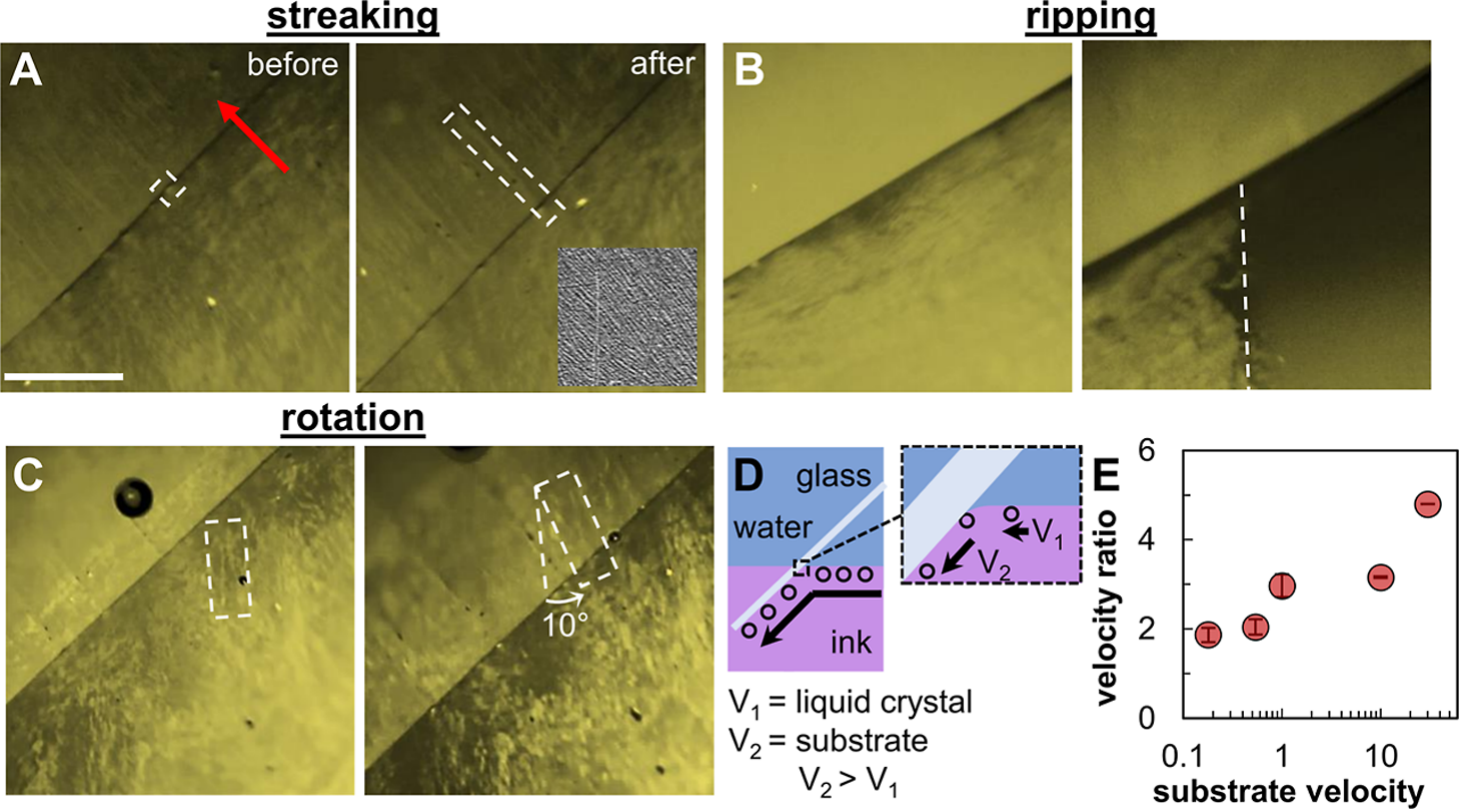
Before and after LC transfer images showing streaking (A), ripping (B), and rotation (C) of LC features during deposition. The scale bar in (A) is 500 μm and applies to all POM images. (D) Cross-sectional schematic of the interface shape and contact line during deposition. (E) Velocity ratio (substrate velocity divided by LC velocity at the liquid–liquid interface) as a function of substrate velocity. Units of substrate velocity are mm min^–1^. The SEM inset in (A) is of CNTs within a streak and is 1.2 μm wide. The red arrow in (A) depicts direction of substrate translation.

A schematic illustrating this discrepancy is provided in Figure 6E. The velocity of the LC features at the interface is measured from the POM videos, and the substrate velocity is set by a motorized linear stage. Increasing the substrate velocity from 0.18 mm min^–1^ to 30 mm min^–1^ increases the velocity mismatch (substrate to LC) from 1.8 to 4.8, as shown in Figure 6D. A similar velocity mismatch was observed in previous studies of the flow field near dewetting contact lines.^30–32^ As the contact line slides along the substrate, a split-streamline flow pattern may develop, inducing flow toward the contact line at the interface. The interfacial velocity depends on the dynamic contact angle, which varies with substrate velocity.^33^ Additionally, flow may be further complicated by surface tension gradients arising from the diffusion of chloroform and/or ethanol into the water. Nevertheless, across the tested conditions, the flow at the interface was consistently directed toward the contact line. While the precise optimal substrate velocity for faithful transfer of CNT LC domains depends on interface conditions and flow profiles, our data indicate that domain deformation increases as the ratio of substrate velocity to interfacial LC velocity exceeds ∼2–3 (Figure 6E). For practical applications, maintaining substrate speeds comparable to the transport speed of the interfacial LC is likely to minimize streaking, tearing, and rotation.

## Conclusions

In this work, we directly confirm that CNTs spontaneously form two-dimensional LC phases at chloroform/water interfaces and demonstrate that this ordering occurs before deposition onto a substrate. By leveraging *in situ* POM, we track the emergence, domain structure, and interfacial dynamics of these CNT LCs, showing how their formation is influenced by factors like CNT concentration and ethanol-induced Marangoni flow. Our results reveal that interfacial assembly is robust across different polymer wrappers and confirms that, under optimized conditions, the LC domain structures are largely retained during transfer. However, distortions during deposition arise when there is a mismatch between substrate velocity and the transport speed of the interfacial LC. These findings provide the first real-time evidence of LC formation during liquid–liquid interfacial CNT assembly. While this study specifically used CNTs of 1.5 and 500 nm in diameter and length, respectively, the LC phenomena reported here should translate to CNTs of other diameters and lengths, provided that the CNT persistence length ≫ CNT length. While the persistence length generally rapidly decreases with CNT diameter, reported CNT persistence lengths of CNTs with a diameter as small as 0.8 nm still exceed 10 μm.^19^ Thus, CNTs fabricated by other methods, e.g., by CoMo catalysis (∼0.8 nm in diameter) or high pressure carbon monoxide catalysis (∼0.8–1.3 nm in diameter), should still remain in the rigid or semiflexible rod regimes, provided their length is a few μm or shorter. Theoretical treatments of semiflexible rods indicate that while flexibility shifts the isotropic–nematic transition to higher particle area densities, nematic ordering still occurs.^34,35^ Indeed, the self-assembly and alignment of CNTs as small as 0.76 nm in diameter have been observed previously at liquid–liquid interfaces.^12,36^

Looking forward, the use of *in situ* POM will be instrumental in developing and analyzing strategies for improving alignment and reducing defects. For example, in tangential flow interfacial self-assembly (TaFISA),^13^ a tangential flow is thought to drive the unidirectional alignment of LC domains—although there are defects. *In situ* POM should provide a means for fine-tuning this method or others to produce dense, uniformly aligned CNT arrays, accelerating progress toward scalable, high-performance CNT-based electronic devices.

## Supplementary Material











## Notes

Calculation of isotropic–nematic phase transition for CNTs in 2D; forward and reverse POM images of a sessile droplet under control conditions; POM images of CNT LCs formed at varying CNT ink concentrations; and POM images of CNT LCs formed using triblock copolymer wrapped CNT ink and PCz-wrapped CNTs dispersed in 1,1,2-trichloroethane (PDF)
POM still images during LC formation (MP4)
POM still images during LC formation (MP4)
Recolored POM movie of CNT LC formation; recolored POM movie of CNT LC formation with Marangoni flow; and black and white particle tracking velocimetry movie in a sessile droplet (MP4)
Recolored POM movie of CNT LC deposition onto a glass substrate; explanation of image and video recoloring algorithm; and table of contrast multiplier and brightness offset values used for image and video recoloring (MP4)
