# Supplementary material for: In Situ Microscopy of 2‑Dimensional Carbon Nanotube Liquid Crystals at Liquid/Liquid Interfaces
Source: Langmuir. 2025 Oct 2;41(40):27393–400. doi: 10.1021/acs.langmuir.5c03535 (PMC12530040; doi:10.1021/acs.langmuir.5c03535)
Supplement: Supplementary file 1 [file la5c03535_si_001.pdf]

Supporting Information for:

In Situ Microscopy of 2-Dimensional Carbon Nanotube Liquid Crystals at Liquid/Liquid Interfaces

James Unzaga[1], Stephanie Oliveras Santos[2], Songying Li[1], Padma Gopalan[1], Arganthaël Berson[3], Michael S. Arnold[1]*

[1] Department of Materials Science and Engineering, University of Wisconsin-Madison, Madison, Wisconsin 53706, United States

[2] Department of Chemistry, University of Wisconsin–Madison, Madison, Wisconsin 53717, United States

[3] Department of Mechanical Engineering, University of Wisconsin–Madison, Madison, Wisconsin 53706, United States

* To whom correspondence should be addressed, michael.arnold@wisc.edu.

Calculation S1.

Calculation of onset of liquid crystal phase for two-dimensional system.

The isotropic-nematic transition for a two-dimensional system is predicted by theory (14) to correspond to an areal density given by:

$$\boldsymbol{\sigma} \approx \frac{\mathbf{7}}{\boldsymbol{L}^{\mathbf{2}}}$$

where *L* is the nanotube length. Using a nanotube length of 500 nm, the areal density at the isotropic-nematic transition is 28 μm^{-2}.

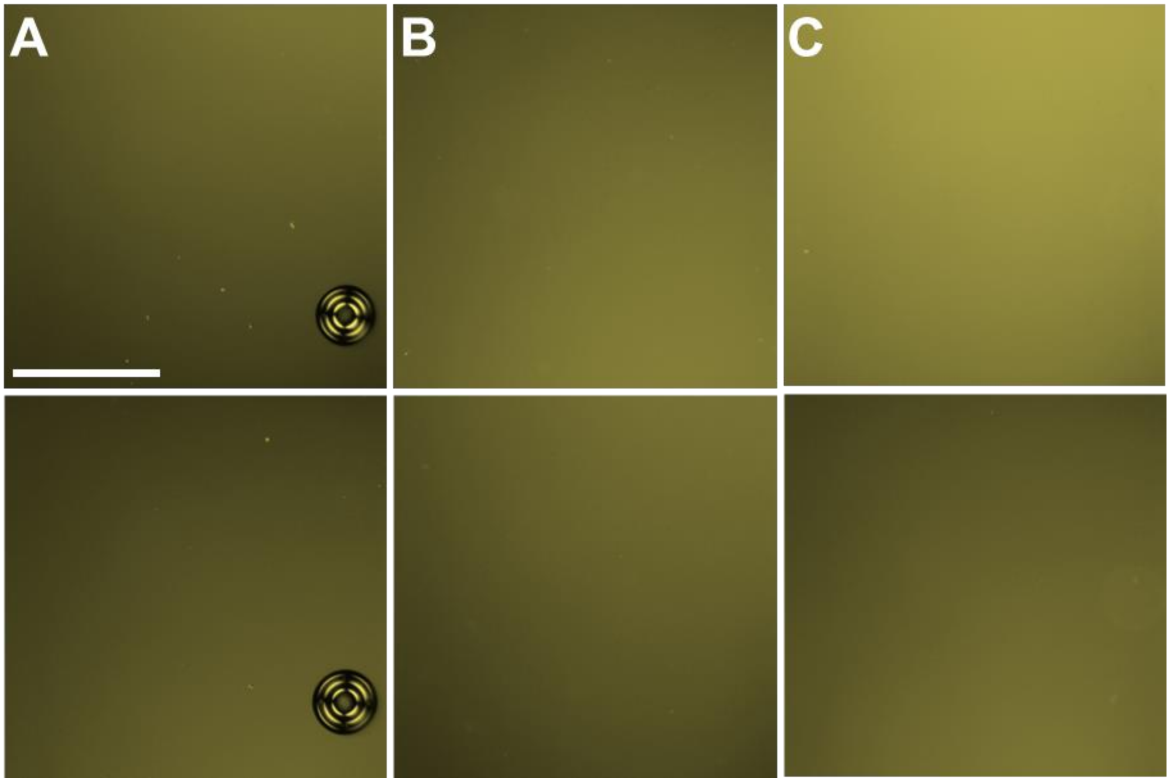

Figure S1. POM images of control conditions demonstrating no LC formation at interface for (**A**) chloroform, (**B**) wrapping polymer (PFO-BPy) and chloroform, and (**C**) ethanol and chloroform mixture. The bottom right of the chloroform case includes a bubble at interface. Scale bar in (**A**) is 500 µm and applies to all images.

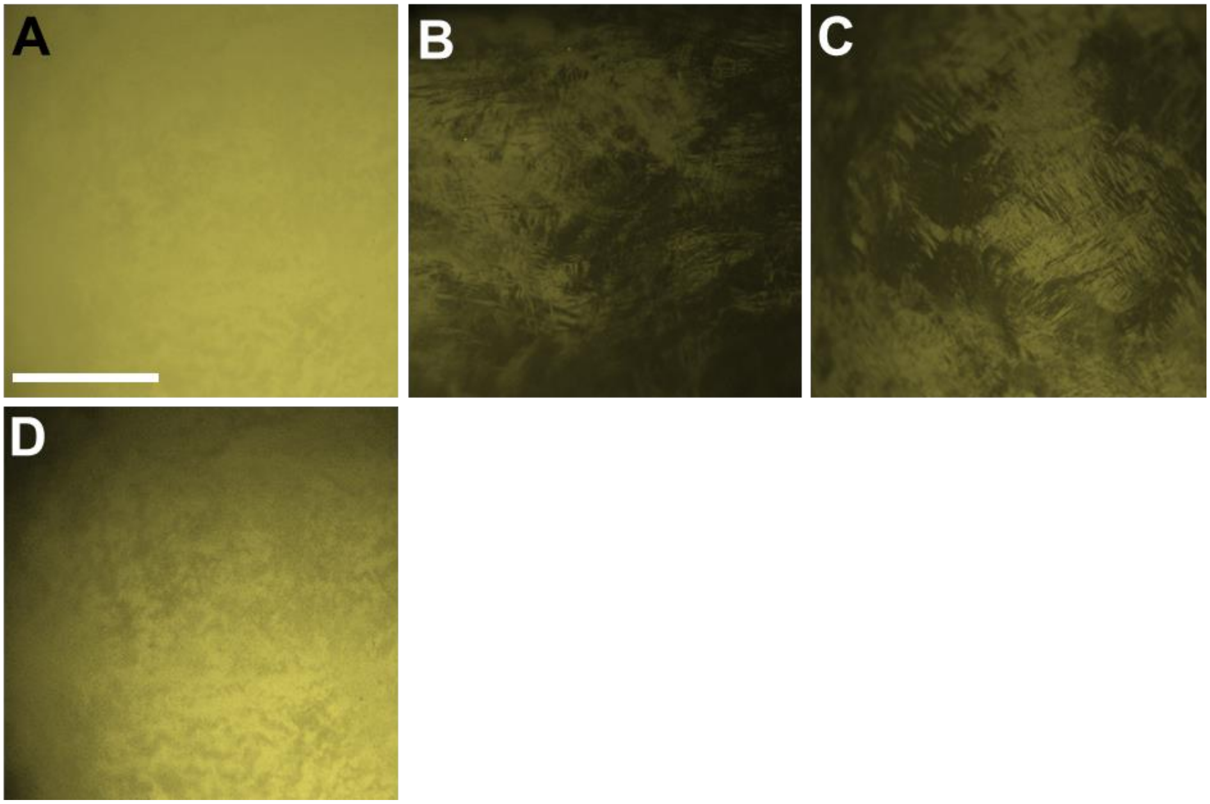

Figure S2. Comparison of liquid crystals formed from (**A**) 5, (**B**) 80, and (**C**) 240 μg/ml CNT ink to illustrate increased CNT density of LC at higher ink concentrations. All three images are taken at the same brightness / contrast. A second POM image of part (**A**) with increased contrast is also provided to better evidence the presence of the CNT LC at the interface (**D**). The scale bar is 500 μm and applies to all images.

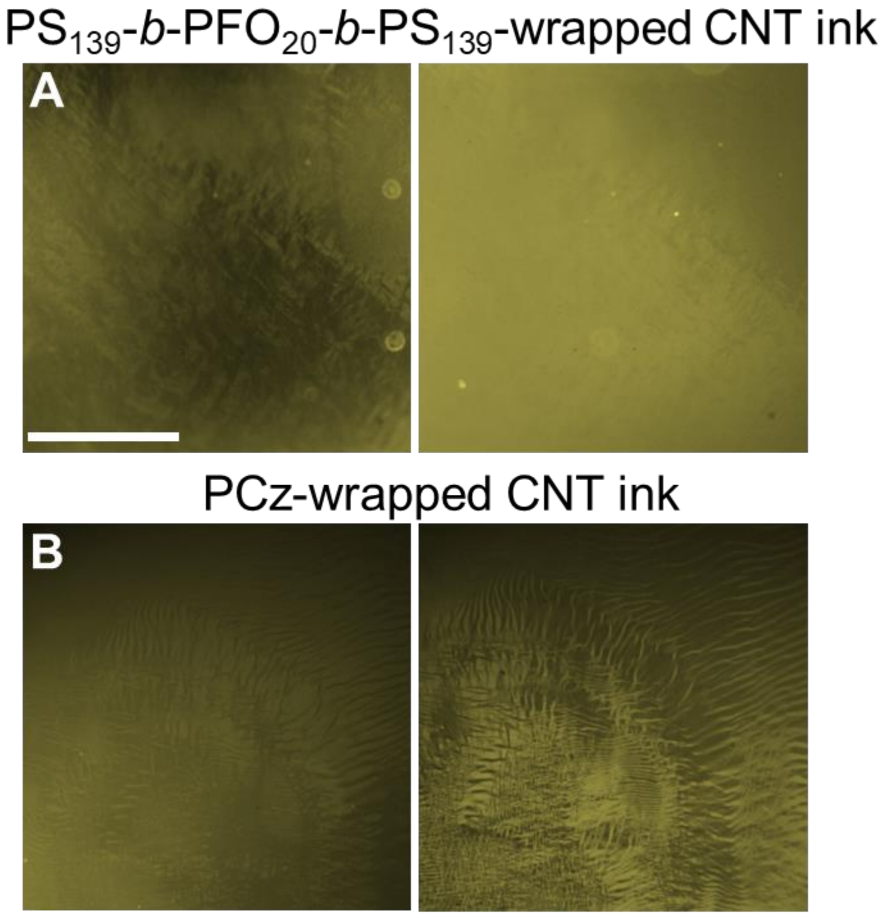

Figure S3. POM images of CNT liquid crystals formed using alternative wrapping polymers: (**A**) PS_{139}-*b*-PFO_{20}-*b*-PS_{139} triblock copolymer and (**B**) polycarbazole. Left and right images are forward and reverse polarizations. The triblock ink was dispersed in chloroform, and part A characterizes the CNTs at the interface of a droplet of this triblock CNT ink injected into a water bath. The polycarbazole ink was dispersed in 1,1,2-trichloroethane, and part B characterizes the CNTs at the interface of a droplet of this polycarbazole CNT ink injected into a bath of 2-butene-1,4-diol. The scale bar in A is 500 μm and applies to all images.

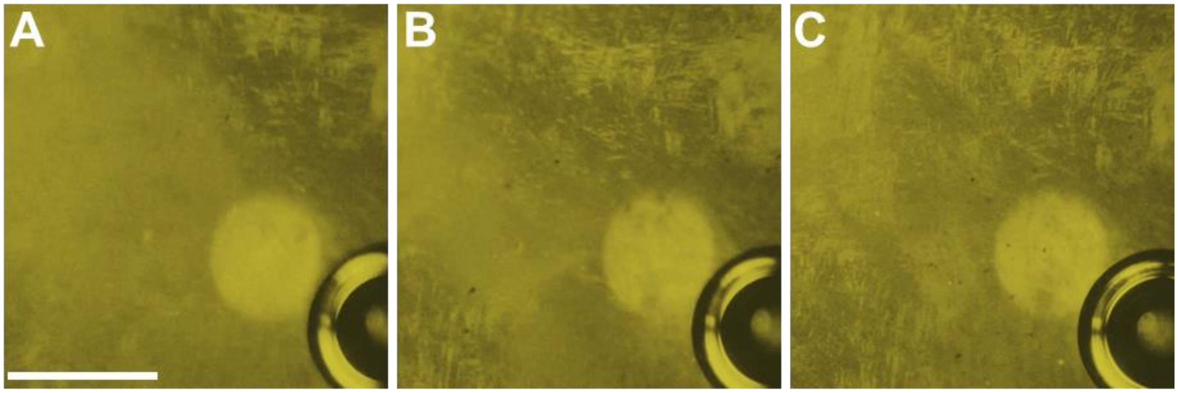

Figure S4. POM images taken 20, 25 and 30 s after the introduction of a droplet of CNT ink into a water bath using 80 µg/ml CNT ink with 1.4% ethanol. The scale bar is 500 µm and applies to all images. These still frames are taken from Video S1.

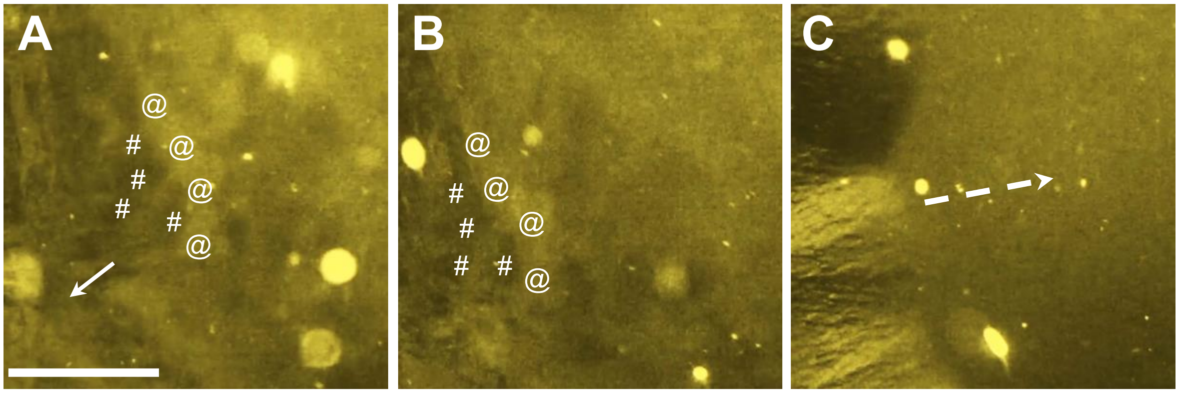

Figure S5. POM images taken 11, 13, and 56s after the introduction of a droplet of CNT ink into a water bath using 80 µg/ml CNT ink with 5% ethanol. LC domains in **A** and **B** are labeled with symbols to highlight movement. The scale bar in **A** is 500 µm and applies to all images. These still frames are taken from Video S2.

Video S1. A recolored video of sequential POM images showing the dynamics of a 2D-LC of CNTs forming at the liquid-liquid interface using 80 µg/ml CNT ink with 1.4% ethanol. Video is played back at 30 frames per second.

Video S2. A recolored video of Marangoni-flow directed growth of a CNT liquid crystal on the surface of a sessile droplet using 80 µg/ml CNT ink with 5% ethanol. Silicon particles are included in the bulk of ink to visualize inward flow. Video is played back at 30 frames per second.

Video S3. Black and white particle tracking velocimetry video of silicon particles suspended within bulk of ink flowing outward towards edges of droplet. Colored dots in video are moving particles measured by PTV open source TracTrac software. Video is played back at 30 frames per second.

Video S4. A recolored video of sequential POM images showing the deposition and transfer of a CNT liquid crystal film onto a glass substrate at a 3 μm sec^{-1} substrate speed. Frames were taken every 5 seconds.

Calculation S2. T = 0.3, k = 14.

Image Transformation Methodology

Given an input color image $\mathbf{I}_{\text{RGB}}(x, y) = [R(x, y), G(x, y), B(x, y)]$, the transformed recolored image $\mathbf{C}_{\text{out}}(x, y)$ is computed by the following steps:

1. Grayscale Conversion

Convert to grayscale by averaging the RGB channels:

$$I(x, y) = \frac{R(x, y) + G(x, y) + B(x, y)}{3}$$

where $I(x, y) \in [0, 1]$.

2. Brightness Adjustment

Add brightness offset γ_b:

$$I_{\text{bright}}(x, y) = \text{clip}\big(I(x, y) + \gamma_b,\ 0,\ 1\big)$$

where clip limits values to $[0, 1]$.

3. Gamma Correction

Apply gamma correction with parameter γ:

$$I_\gamma(x, y) = \big(I_{\text{bright}}(x, y)\big)^\gamma$$

4. Soft Thresholding

Calculate weight $w(x, y)$ via sigmoid:

$$w(x, y) = \frac{1}{1 + \exp\big(k \cdot (I_\gamma(x, y) - T)\big)}$$

with threshold $T \in [0, 1]$ and steepness $k > 0$.

5. Color Interpolation

Define custom colors:

$$\mathbf{C}_{\text{black}} = (R_b, G_b, B_b), \quad \mathbf{C}_{\text{yellow}} = (R_y, G_y, B_y)$$

For the specific colors used:

$$\mathbf{C}_{\text{black}} = (0.157, 0.149, 0.067), \quad \mathbf{C}_{\text{yellow}} = (0.929, 0.878, 0.388)$$

Interpolate final pixel color:

$$\mathbf{C}_{\text{out}}(x, y) = w(x, y) \cdot \mathbf{C}_{\text{black}} + (1 - w(x, y)) \cdot \mathbf{C}_{\text{yellow}}$$

Table S1. Table of contrast multiplier and brightness offset values for figures and videos included.

Figure Panel	α (contrast multiplier)	γ (brightness offset)
Figure 1 B	1.60	0.20
Figure 2 A	1.60	0.00
Figure 2 B	1.60	0.00
Figure 3 A	1.00	0.00
Figure 3 B	1.00	0.00
Figure 3 C	1.00	0.00
Figure 4 A	1.65	0.20
Figure 4 B	1.65	0.20
Figure 4 C	1.65	0.20
Figure 4 D	1.65	0.20
Figure 5 B	1.00	0.40
Figure 5 C	1.00	0.40
Figure 5 D	1.00	0.40
Figure 6 A L	0.85	0.30
Figure 6 A R	0.85	0.30
Figure 6 B L	1.00	0.20
Figure 6 B R	1.00	0.40
Figure 6 C L	1.35	0.45
Figure 6 C R	1.65	0.35
Video S1	1.10	0.00
Video S2	1.10	0.00
Video S4	1.00	0.00
Figure S1	1.00	0.00
Figure S2 ABC	1.40	0.00
Figure S2 D	1.80	-0.10
Figure S3 A	1.00	0.30
Figure S3 B	1.10	0.00
Figure S4	1.25	-0.20
Figure S5 A	1.30	0.10

Figure S5 B	1.30	0.10
Figure S5 C	1.30	0.10
